# Quantum Information and Computation

**DOI:** 10.3390/e25030463

**Published:** 2023-03-07

**Authors:** Shao-Ming Fei, Ming Li, Shunlong Luo

**Affiliations:** 1 School of Mathematical Sciences, Capital Normal University, Beijing 100048, China; 2 School of Science, China University of Petroleum, Qingdao 266580, China; 3 Academy of Mathematics and Systems Science, Chinese Academy of Sciences, Beijing 100190, China

Quantum technology can break through the bottleneck of traditional information technology by ensuring information security, speeding up computation, improving measurement accuracy, and providing revolutionary solutions to some issues of economic and social development. The theory of quantum information and computation provides guarantee on the development of quantum technology. This Special Issue is intended to investigate some basic features and applications of quantum information, including but not limited to complementarity, quantum algorithms, quantum coherence, quantum correlations, quantum measurement, quantum metrology, quantum uncertainties, and quantum information processing.

The works in this Special Issue can be divided into two categories: basic theory of quantum information, and quantum information processing and algorithm designs. We start with the former.

A quantum channel usually changes the quantum features of the system, such as causing decoherence of quantum states and destroying quantum correlations. Characterizing quantum channels from the information perspective has yielded fruitful results. In [1], Song and Li propose a framework to qualitatively and quantitatively characterize quantum channels from the perspective of the amount of quantumness in ensembles that a quantum channel can induce. They investigate the dynamics of quantumness in ensembles and propose quantumness power and dequantumness power to characterize quantum channels. If a channel reduces the quantumness for all the ensembles at all times, it is a completely dequantumness channel. The relationship with Markovian channels is also studied through several examples. The work illustrates new properties of quantum channels from the perspective of the information flow in terms of quantumness brought by the interaction between the system and environment. The results can be directly generalized to arbitrary dimensions and other measures of quantumness.

Quantum verification has been highlighted as a significant challenge on the road to scalable technology. In addition to the tomography of a quantum state, self-testing is a device-independent approach to verifying that the previously unknown quantum system state and uncharacterized measurement operators are, to some degree, close to the target state and measurements (up to local isometries) based only on the observed statistics, without assuming the dimension of the quantum system. Previous studies focused on bipartite states and some multipartite states, including all symmetric states, but only in the case of three qubits. Bao et al. [2] give a criterion for the self-testing of a four-qubit symmetric state with a special structure and provide a robustness analysis based on vector norm inequalities. Bao et al. also generalize the idea to a family of parameterized four-qubit symmetric states through projections onto two subsystems.

The Belavkin–Staszewski (BS) relative entropy is an enticing and crucial entropy used to process quantum information tasks, which can be used to describe the effects of possible noncommutativity of the quantum states (the quantum relative entropy does not work well for this case). Katariya and Wilde employed the BS relative entropy to study quantum channel estimation and discrimination. Bluhm and Capel contributed a strengthened data processing inequality for BS relative entropy. This property was first established by Hiai and Petz. Bluhm et al. presented some results on weak quasi-factorization for BS relative entropy. Fang and Fawzi studied quantum channel capacities with respect to the geometric Rényi relative entropy. In [3], Zhai et al. define two new conditional entropy terms and four new mutual information terms by replacing quantum relative entropy with BS relative entropy. Some basic properties of the newly introduced entropies are investigated, especially in classical-quantum settings. In particular, the authors of [3] show the weak concavity of the BS conditional entropy and obtain the chain rule for the BS mutual information. Finally, the subadditivity of the BS relative entropy is established; i.e., the BS relative entropy of a joint system is less than the sum of its corresponding subsystems with the help of some multiplicative and additive factors. Meanwhile, a certain subadditivity of the geometric Rényi relative entropy is also provided.

One of the fundamental phenomena in quantum physics is the impossibility of simultaneous realization of two quantum operations. The Heisenberg uncertainty principle and the no-cloning theorem are two famous incarnations for such phenomena. Generally, two (or more) quantum operations, such as measurements, channels, or instruments, are called compatible if they can be seen as the marginals of a common operation. Otherwise, they are called incompatible. The concept of incompatibility of quantum channels has been proposed in terms of the input–output devices. In [4], Zhang and Nechita present a new incompatibility criterion for quantum channels based on the notion of (quantum) Fisher information. The power of this incompatibility criterion is further discussed in different scenarios. The authors of [4] present the analytical conditions for the incompatibility of two Schur channels. They also investigate the incompatibility structure of a tuple of depolarizing channels by comparing the newly introduced criterion with the known results from asymmetric quantum cloning.

As an important resource in many quantum information tasks, quantum coherence is a feature of quantum systems rooted in the superposition principle. Since the coherence of quantum states depends on the choice of the reference basis, it is natural to study the relationship among the coherence with respect to different bases. Wang et al. [5] study quantum incoherence based simultaneously on k bases. They firstly define a correlation function m(e,f) of two orthonormal bases e and f, by which the relationships between sets I(e) and I(f) of incoherent states are investigated. They show that I(e) = I(f) if and only if the rank-one projective measurements generated by e and f are identical. They also provide a necessary and sufficient condition for the intersection I(e)⋂I(f) to include a state except the maximally mixed state. In particular, if two bases e and f are mutually unbiased, then the intersection has only the maximally mixed state. The authors then introduce the concepts of strong incoherence and weak coherence of a quantum state with respect to a set B of k bases and propose a measure for the weak coherence. They prove that in two-qubit systems there exists a maximally coherent state with respect to B for k = 2 but not for k = 3.

Entanglement is a quintessential manifestation of quantum mechanics and is often considered to be a useful resource for tasks such as quantum teleportation or quantum cryptography. Genuine multipartite entanglement is an important type of entanglement that offers significant advantages in quantum tasks compared to bipartite entanglement. The distribution of entanglement is believed to be monogamous, i.e., a quantum system entangled with another system limits its entanglement with the remaining others. There are two methods used in this research. The first one is to analyze monogamy relations based on bipartite entanglement measure, and the second one is based on multipartite entanglement measure. In [6], Guo explores the complete monogamy of a genuine multipartite entanglement measure (GMEM). Guo firstly studies the framework for unified/complete GMEM according to the unified/complete multipartite entanglement measure. He finds a way of inducing unified/complete GMEM from any given unified/complete multipartite entanglement measure. It is shown that any unified GMEM is completely monogamous, and any complete GMEM that is induced by the given complete multipartite entanglement measure is completely monogamous. In addition, the previous GMEMs are checked under this framework. It turns out that the genuinely multipartite concurrence is not a good candidate for GMEM.

Entanglement detection is a basic problem in quantum theory. A powerful tool is the so-called positive partial transpose (PPT) criterion, proposed by Peres. PPT condition is not only necessary but also sufficient for the separability of qubit–qubit, qubit–qutrit, or qutrit–qubit systems. Rana shows that all eigenvalues of any partially transposed bipartite state fall within the closed interval [−1/2, 1]. In [7], Duan et al. study a family of bipartite quantum states for which the minimal eigenvalues of the partially transposed states are −1/2. For a two-qubit system, the authors of [7] find that the minimal eigenvalue of its partially transposed state is −1/2 if and only if the two-qubit state is maximally entangled. They also show that this result does not hold in general for two-qubit systems when the dimensions of the underlying space are larger than two.

The second category of the works in this Special Issue focuses on quantum information processing and algorithm designs. 

Since the multi-locality in quantum networks features several independent sources under joint measurements, one can obtain stronger correlations throughout the whole network. Such networks were first observed in a bi-local network. Since then the nonlocality of various quantum networks has been explored, including chain-shaped networks, star-shaped networks, triangle networks, and tree-tensor networks. In [8], Yang et al. consider the nonlocality of any forked tree-shaped networks, where each node shares an arbitrary number of bipartite sources with other nodes in the next “layer”. Yang et al. derive Bell-type inequalities for such quantum networks in terms of all $\left(t_{n}-1\right)$-local correlations and all local correlations, where $t_{n}$ denotes the total number of nodes in the network. The authors of [8] also derive the maximal quantum violations of these inequalities and the robustness to noise in these networks.

In recent years, quantum computing has been extensively studied from theory to practice. Noisy intermediate-scale quantum (NISQ) computers may not have the capability to deal with large-scale quantum information processing. Delegating computations to the companies that offer quantum computing may be a better choice to access quantum computers. In [9], Ma et al. propose a distributed secure delegated quantum computation protocol, which allows clients to delegate their private computation to several quantum servers. Ma et al. show that their protocol can guarantee unconditional security of the computation under the situation where all servers, including the third party, are honest but curious, and they are allowed to cooperate with each other.

As an important application of quantum theory, quantum key distribution (QKD), which allows two users to share a secure key privately, is one of the options to combat the lack of safety in communication caused by the increasingly developed quantum computation. Bennett and Brassard present the first QKD protocol. In [10], Fan et al. propose a design rule of rate-compatible low-density parity-compatible codes which covers all potential signal-to-noise ratio with a single check matrix. By such codes, high-efficiency continuous-variable quantum key distribution information reconciliation is achieved.

There exist various loopholes in practical systems through which eavesdroppers can attack the QKD process. Lo et al. present a measurement-device-independent quantum key distribution (MDI-QKD) protocol to prevent attacks on measurement devices and enhance the communication distance between two users. In [11], Hua et al. propose a flexible multi-user measurement-device-independent quantum key distribution (MDI-QKD) scheme based on a GHZ entangled state. Hua’s scheme can distribute quantum keys among multiple users while being resistant to detection attacks. Hua et al. then present simulation results which show that the secure distance between each user and the measurement device can reach more than 280 km, while reducing the complexity of the quantum network. Hua et al. also present a method to expand the scheme to a multi-node, multi-user network, which can further enhance the communication distance between the users at different nodes.

In quantum information processing, errors are inevitable. Quantum error-correcting codes (QECCs) are invented to ensure the implementation of quantum communication and quantum computing. Pang et al. [12] draw support from the Hamming distance and minimal distance of orthogonal arrays to study the relationship between uniform states and binary QECCs. They provide new methods to construct pure quantum error-correcting codes. By using these methods, several infinite series of quantum error-correcting codes including some optimal ones are constructed. 

A large number of practical problems often boil down to solving a system of linear algebraic equations, such as elasticity in engineering and science, circuit analysis, geodesy, heat conduction, vibration, etc. Many effective numerical methods, such as spline interpolation, least-squares fitting, generalized Newton method for solving nonlinear equations, method for calculating the coefficients of Newton–Cotes quadrature formula, finite difference method, and finite element method for solving numerical solutions of partial differential equations, are also finally converted into problems of solving linear equations. Zhang et al. in [13] provide a modified quantum scheme to obtain the quantum state corresponding to the solutions of linear system of equations with less machine running time than the existing quantum algorithms. The authors of [12] also investigate the problem of finding solutions to a linear system with a sparsity-independent and non-square coefficient matrix.

Quantum entanglement swapping is a highly significant technology in quantum entanglement repeaters, which are generally employed to realize long-distance quantum entanglements in many quantum information processing tasks. In [14], Xie et al. discuss the problem of quantum correlation (QC) swapping between two Werner-like states by performing Bell measurements on the middle node and taking into account the measurement-induced disturbance (MID) and ameliorated MID.
